# How Should Home-Based Maternal and Child Health Records Be Implemented? A Global Framework Analysis

**DOI:** 10.9745/GHSP-D-19-00340

**Published:** 2020-03-30

**Authors:** Sruthi Mahadevan, Elena T. Broaddus-Shea

**Affiliations:** a University College London Medical School; Royal Free London NHS Foundation Trust, London, UK.; b Department of Family Medicine, University of Colorado Anschutz Medical Campus, Aurora, CO, USA.

## Abstract

**Background:**

A home-based record (HBR) is a health document kept by the patient or their caregivers, rather than by the health care facility. HBRs are used in 163 countries, but they have not been implemented universally or consistently. Effective implementation maximizes both health impacts and cost-effectiveness. We sought to examine this research-to-practice gap and delineate the facilitators and barriers to the effective implementation and use of maternal and child health HBRs especially in low- and middle-income countries (LMICs).

**Methods:**

Using a framework analysis approach, we created a framework of implementation categories in advance using subject expert inputs. We collected information through 2 streams. First, we screened 69 gray literature documents, of which 18 were included for analysis. Second, we conducted semi-structured interviews with 12 key informants, each of whom had extensive experience with HBR implementation. We abstracted the relevant data from the documents and interviews into an analytic matrix. The matrix was based on the initial framework and adjusted according to emergent categories from the data.

**Results:**

We identified 8 contributors to successful HBR implementation. These include establishing high-level support from the government and ensuring clear communication between all ministries and nongovernmental organizations involved. Choice of appropriate contents within the record was noted as important for alignment with the health system and for end user acceptance, as were the design, its physical durability, and timely redesigns. Logistical considerations, such as covering costs sustainably and arranging printing and distribution, could be potential bottlenecks. Finally, end users' engagement with HBRs depended on how the record was initially introduced to them and how its importance was reinforced over time by those in leadership positions.

**Conclusions:**

This framework analysis is the first study to take a more comprehensive and broad approach to the HBR implementation process in LMICs. The findings provide guidance for policy makers, donors, and health care practitioners regarding best implementation practice and effective HBR use, as well as where further research is required.

## Abstract (precis)

Our assessment of home-based record use in low- and middle-income countries indicated that the implementation process consists of 8 interdependent components involving policy makers, funders, and end users—health care workers, pregnant women, and the parents/caregivers of children. Successful implementation can result in improved maternal and child health outcomes and more efficient use of government and donor investments.

## INTRODUCTION

A home-based record (HBR) is a physical or electronic health document that a patient or caregiver keeps, rather than a health facility.^1^ HBRs are used by pregnant women and parents/caregivers of children as an instant access to their own or their child's health information, by health care workers and volunteers to triangulate patient information and to prompt health education conversations, and by public health administrators as a data management tool to help plan and evaluate services.^2^

HBRs were initially introduced in immunization programs but have since become widely used throughout maternal, neonatal, and child health (MNCH) programs.^3^ They can take many forms including antenatal care records, vaccination-only cards, vaccination-plus cards or booklets, child health books, and combined maternal and child health books.^1^^–^^4^ They are designed to record health parameters such as developmental milestones, vaccinations received, or antenatal appointments attended. Some HBRs also contain health education messages, for example, about immediate and exclusive breastfeeding or managing minor childhood illnesses.^3^^,^^5^ Throughout this article, we use the term HBR to encapsulate the diversity of different record types.

This study was part of the evidence synthesis for the World Health Organization (WHO) recommendations on HBRs for MNCH^3^ alongside a quantitative systematic review and meta-analysis of peer-reviewed literature^6^ and a qualitative review of peer-reviewed literature.^7^ The guidelines, published in September 2018, recommend HBRs as a tool to improve MNCH outcomes.^3^ Studies summarized within the quantitative review and meta-analysis found that HBRs had statistically significant effects on improving antenatal care attendance and supportive household environments; however, the evidence base is small and of low to very low certainty.^6^^,^^8^ Further effects, also of low certainty, were seen for child health including immunization completion, infant feeding, growth and development monitoring, and reducing risks of cognitive delay.^6^ Even though the effects seen on individual end users in each study were small, the WHO deemed that HBRs in MNCH still have the potential to create significant large-scale impact since the population of end users is so vast.^3^ Future research is needed to further explore impacts on each health outcome and potential undesirable effects since no harms were identified from the available evidence, but there was a paucity of evidence on many of the outcomes studied.^3^^,^^6^

Despite less quantitative data on MNCH outcomes, there were significant data from the qualitative and quantitative studies, as well as from the respondents in this study, that emphasized that women, caregivers, and health care workers value HBRs.^3^^,^^6^^,^^7^ There is evidence that they can improve patient-provider communication, continuity of care,^7^^,^^9^ and increase women's feelings of control and empowerment.^7^^,^^10^ Including and empowering women and children by encouraging them and their caregivers to be health literate and actively participate in their own care is a key step toward achieving universal health coverage.^11^

Unfortunately, despite being underpinned by research, many evidence-based interventions never gain traction in routine practice or they only do so after many years.^12^ HBRs can be found in at least 163 countries; however, population coverage varies wildly between these countries and even between regions within countries.^13^ For example, in Bangladesh, Ethiopia, Indonesia, Nigeria, and Pakistan—countries with some of the world's highest birth rates—less than 50% of eligible mothers and children owned HBRs.^13^ Even where HBRs were available, they were not always used fully or as they were intended.^14^ Implementation science has proved that focusing on and optimizing the implementation of health tools and services improves equity of coverage and improves quality and efficiency.^12^^,^^15^ By optimizing implementation of HBRs, we can potentially increase ownership and equity of coverage and improve engagement with the HBR content. By doing so, we can also maximize value from limited resources and maximize overall public health impact.

Optimizing implementation of HBRs can potentially increase ownership and equity of coverage and improve engagement with the HBR content.

It is difficult to recommend strategies to improve HBR implementation without first identifying the blockages along the implementation pipeline that have resulted in this research-to-practice gap. Existing literature has examined selected aspects of implementation, some slightly more than others, namely design of the HBR^2^^,^^16^^–^^20^ and promotion of end user engagement.^21^^–^^24^ However, substantial operational challenges still remain even in these areas and require further research.^14^^,^^25^ The majority of existing literature studied HBRs in high-income countries.^3^^,^^6^^,^^7^ This study sought to identify a broad understanding of the HBR implementation process in low- and middle-income countries (LMICs) and the possible facilitators and barriers to each implementation component. What are the causes of ineffective implementation, and why do they persist? Conversely, how do we build successful HBR programs?

## METHODS

Framework analysis is a qualitative thematic analysis approach that involves an ongoing and dynamic interplay between textual data collection, analysis, and theory development. A matrix of structured summarized data is created and categorized by key themes to identify similarities and differences both between and within the data from each data source.^26^ Therefore, it is suited to the dual data sources used for this study: semi-structured interviews and written documents that have been prescreened for relevance. The sampling of data sources was designed to capture the diversity of responses around each key theme and was not intended to proportionately represent HBRs according to the prevalence of each different HBR type.^26^

### Framework Development

The WHO's HBR Working Group comprised a small group of HBR experts who worked for the past few years assisting national-level ministries and nongovernmental organizations to effectively redesign their HBRs. These subject experts created an initial framework of expected implementation components that consisted of 15 categories. We used the initial framework to develop our interview guide. During each interview, we also delved further into any emerging themes and explicitly asked respondents whether they felt any other implementation components deserved discussion. After analyzing the collected data, we iteratively revised the framework to remove less relevant themes or subsume them under broader categories and to include new themes and categories emerging from the data. The initial and revised frameworks are included in a Supplement.

### Data Collection

Data used in this analysis consisted of semi-structured key informant interviews and relevant gray literature documents.

#### Key Informant Interviews

We contacted 25 individuals with experience in HBR implementation in LMICs. Of these, 13 replied and indicated willingness to be interviewed. Due to adverse weather events in 1 respondent's country, we conducted 12 interviews using phone or Skype. Key informants included individuals with associations to the Japan International Cooperation Agency (n=2), John Snow, Inc. (n=5), United Nations Relief and Works Agency for Palestine Refugees in the Near East (n=1), and United Nations Population Fund (n=4). They had diverse professional expertise including a mix of international (n=6) and in-country (n=6) work, a combination of frontline service provision and management perspectives, and a variety of MNCH specializations (Table 1).

**TABLE 1.** Characteristics of Key Informants Who Were Interviewed About HBR Implementation (N=12)

Number	Job Level	Qualifications	HBR Type Familiar With	Countries Familiar With
R1	International	Maternal and Child Health Program Expert	Multiple focus	Afghanistan, Angola, Burundi, Cambodia, Cameroon, China, Gabon, Ghana, India, Indonesia, Kenya, Lao PDR, Micronesia, Myanmar, Palestine, Philippines, Rwanda, Senegal, Tajikistan, Thailand, Vietnam, Timor Leste, Uganda
R2	International	Maternal and Child Health Program Expert	Multiple focus	Gaza, Jordan, Lebanon, Syria, West Bank
R3	Country	Maternal and Child Health Program Expert	Multiple focus	Madagascar
R4	Country	Maternal and Child Health Services Expert	Single focus	Bangladesh (current), India, Rwanda, Somalia, Sierra Leone
R5	Country	Maternal and Child Health Program Expert	Single focus	Nepal
R6	International	Maternal and Child Health Program Expert	Multiple focus	Ghana, Jordan, Lebanon, Palestine, Syria
R7	Country	Midwifery Specialist	Multiple focus	Pakistan
R8	Country	Midwifery Specialist	Multiple focus	Zambia
R9	Country	Midwifery Specialist	Multiple focus	Ethiopia (current), Malawi
R10	International	Senior Immunization Technical Officer	Single and multiple focus	Benin, Cameroon, Democratic Republic of the Congo, Ghana, India, Kenya, Madagascar, Nepal, Nigeria, Tanzania, Zimbabwe
R11	International	Maternal and Child Health Program Expert	Single and multiple focus	Ethiopia, Ghana, Liberia, Madagascar
R12	International	Immunization Program Expert	Single and multiple focus	Bangladesh, Ethiopia, Madagascar

Abbreviation: HBR, home-based record.

Each interview was conducted by 2 members of the research team using an interview guide based on the initial analytic framework categories. Detailed notes were taken on respondents' answers, interviews were recorded with permission, and recordings were referenced for clarification or further detail as needed. Interviews lasted approximately 45 minutes. Afterward, notes were compiled and sent to the respondent to review for accuracy. Interviews were conducted until it was determined that saturation had been reached on the key topics of interest (i.e., little new information emerged with each interview).

#### Document Review

All interview participants and other staff from organizations involved in HBR implementation were asked to provide relevant program documents, workshop reports, and other gray literature that provided insight and information on the HBR implementation process. Documents were also obtained from the WHO's HBR Working Group. Screening criteria were drafted to include or exclude documents based on relevance to HBR implementation in MNCH. We included documents that addressed at least 1 HBR implementation component, the barriers or facilitators to implementation components, or context-specific aspects of HBR implementation—each with justification of their importance in terms of empirical outcomes or stakeholder perspectives. We excluded documents if they studied the impact that HBRs have on MNCH outcomes but did not address implementation or if they made implementation recommendations without any justification. In total 69 documents were screened. We conducted all screening individually, following initial double-screening of 20 documents that indicated consistency in application of the screening criteria. In total, 18 documents were included in the analysis: technical briefs or reports (n=5) and presentations (n=8), case study (n=1), blog post (n=1), working paper (n=1), newspaper article (n=1), and project proposal (n=1). All documents described implementation in LMICs—a detailed breakdown of the characteristics of all the gray literature documents is in Table 2.

**TABLE 2.** Document Characteristics of Gray Literature Included in Framework Analysis of HBR Implementation

ID	Document Title	Source	HBR Type Addressed	Countries Addressed
D1	Omar MA, Sugushita T. *Kenya: What Mothers Have MCH Booklet?* Tokyo, Japan: Japan International Cooperation Agency; 2016.	JICA	Multiple focus	Kenya
D2	JSI. *Home-Based Record Redesigns That Worked: Lessons from Madagascar & Ethiopia*. Rosslyn, VA: John Snow, Inc; 2017.	JSI	Multiple focus	Madagascar and Ethiopia
D3	Basic Support for Institutionalizing Child Survival Project (BASICS II). *Madagascar Case Study: Improving Family Health Using an Integrated Community-Based Approach*. Arlington, VA: USAID; 2004.	JSI	Multiple focus	Madagascar
D4	John Snow, Inc (JSI). *Country Experiences With Home-Based Records (HBR): Survey Conducted by JSI and the Gates Foundation Jan - Apr 2016.* Presentation at the Home-Based Records Revitalisation Workshop; February 21–24, 2017; Kampala, Uganda.	JSI, Bill & Melinda Gates Foundation	Single and multiple focus	24 different countries in Africa and Asia
D5	Aiga H. *Self-Monitoring Child Nutrition Status Through MCH Handbook*. Tokyo, Japan: Japan International Cooperation Agency, 2013.	JICA	Multiple focus	Vietnam
D6	Rapp A, Hasman A, Radka R. *Home-Based Records Revitalisation Workshop: Workshop Report*. WHO, Bill & Melinda Gates Foundation/Claro; 2016.	Bill & Melinda Gates Foundation, UNICEF, Claro	Single and multiple focus	Afghanistan, India, Nepal, Pakistan
D7	Rane M. Blog Post. www.mrane.com. *Redesigning the Immunization Card for an Indian Context.* 2016.	Indian Institute of Technology Bombay	Single focus	India
D8	WHO/UNICEF/Bill and Melinda Gates Foundation. *Preparation Work Questionnaire Cameroon.* Presentation at the Home-Based Records Revitalisation Workshop; February 21–24, 2017; Kampala, Uganda.	WHO, UNICEF, Bill & Melinda Gates Foundation	Multiple focus	Cameroon
D9	Ministry of Health, Federal Democratic Republic of Ethiopia. *Home-Based Record Revitalization Workshop-Ethiopia*. Presentation at the Home-Based Records Revitalisation Workshop; February 21–24, 2017; Kampala, Uganda.	Ethiopia Ministry of Health	Single focus	Ethiopia
D10	WHO, UNICEF, Bill and Melinda Gates Foundation. *Home-Based Records Revitalisation Workshop: Preparation Work Questionnaire Liberia.* Presentation at the Home-Based Records Revitalisation Workshop; February 21–24, 2017; Kampala, Uganda.	WHO, UNICEF, Bill & Melinda Gates Foundation	Single focus	Liberia
D11	Anya B/WHO Regional Office for Africa. *Home-Based Records Context in the African Region.* Presentation at the Home-Based Records Revitalisation Workshop; February 21–24, 2017; Kampala, Uganda.	WHO, Regional Office for Africa	Single and multiple focus	Regional Office for Africa countries
D12	Gazi R, Khatun J, Ashraf A, ul-Alam M, Kabir H. *Assessment of Retention, Perceived Usefulness, and Use of Family Health Card in the Bangladesh Health and Population Sector Programme*. Dhaka, Bangladesh: ICDDR,B: Centre for Health and Population Research; 2003.	ICDDR,B	Multiple focus	Bangladesh
D13	Kanda A. *Child Health Handbook Put in App for Refugees in Jordan*. The Asahi Shimbun. Asia and Japan Watch - Japan News Section. April 3, 2017.	Japanese National Newspaper	Multiple focus	Jordan
D14	Hagiwara A. *Development of New Combined Maternal and Child Health Record Book in Ghana: Background, Achievement and Way Forwards*. Tokyo, Japan: Japan International Cooperation Agency; 2017.	JICA	Multiple focus	Ghana
D15	Hagiwara A. *What is Maternal and Child Health (MCH) Handbook? Introduction of MCH Handbook to Ghana.* Tokyo, Japan: Japan International Cooperation Agency, 2017.	JICA	Multiple focus	Ghana
D16	Aboagye P, Hodgson A, Ogasawara Y, Hagiwara A. *Progress of Development of MCH Record Book in Ghana*. Poster presented at 10th International Conference on the MCH Handbook; November 23–25, 2016; Tokyo, Japan.	Ghana Health Service, JICA	Multiple focus	Ghana
D17	Hagiwara A/Japan International Cooperation Agency. *MCH Handbook for Refugees.* Presentation at the Home-Based Records Revitalisation Workshop; February 21–24, 2017; Kampala, Uganda.	JICA	Multiple focus	Gaza, Jordan, Lebanon, Syria, West Bank
D18	Service de la vaccination, Ministère de la santé et du planning familial. *Enquête sur la couverture vaccinale*. Antananarivo: Ministère de la santé et du planning familial, Repoblikan'i Madagasikara (Republic of Madagascar); 2008.	Madagascar Ministry of Health	Single focus	Madagascar

Abbrevation: HBR, home-based record; JICA, Japan International Cooperation Agency; JSI, John Snow, Inc.; UNICEF, United Nations Children's Fund; WHO, World Health Organization.

### Data Analysis

As data collection progressed, we took notes on and discussed emerging themes, trends, and other impressions and revised the analytic framework accordingly. Once the majority of data collection was complete, we began extracting information from documents and interviews and summarizing the information into a matrix. Each row in the matrix represented a data source, and each column represented a topic or theme from the revised analytic framework. We both first charted the data from the same 3 sources to check consistency on the interpretation of framework categories, the rest of the documents and interview notes were charted individually. After completing data abstraction, we identified a refined list of 8 key implementation components that best captured the emerging themes.

## RESULTS

We identified 8 main components of the implementation process (Figure). All the components of the implementation process are spokes of the wheel of a successful HBR program. We present the components as a logical way for readers to follow the process, not by importance or true chronological linearity. In practice, these can take place concurrently or nonsequentially.

**FIGURE:**
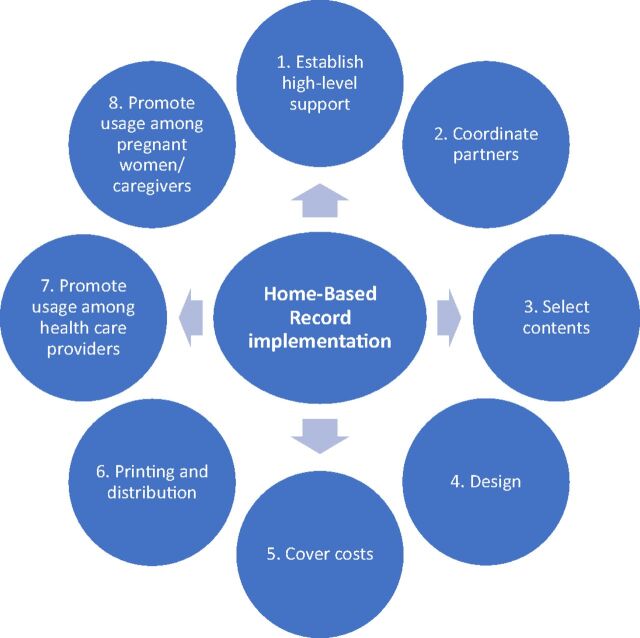
Eight Components of a Successful Home-Based Record Program Implementation Process

### 1. Establishing High-Level Support

*The government can push something that they really want to prioritize.* —Key Informant 4

Many respondents and documents noted that when a government did not take HBRs seriously, it gave less substantial and sustainable support (in terms of time, human resources, and budget prioritization) to the HBR program compared to settings with more committed governments.

Many respondents highlighted that to achieve high-level support, strong advocacy is required to educate government stakeholders, particularly health ministry officials, and donor and coordinating agencies about the benefits of an HBR program and what is required to implement it. It was noted that currently this advocacy came largely from external agencies specializing in HBRs, though it could have also been from responsible officials within the ministries of health.

Respondents noted that strong advocacy is required to educate government stakeholders and donor and coordinating agencies about the HBR program benefits and implementation requirements.

Respondents identified robust evidence about HBRs to be a prerequisite for successful and credible advocacy. Although this seemed to exist in a few country contexts, most respondents were frustrated by a paucity of data on HBRs. A common suggestion was for a question on HBR ownership and usage to be included in national Demographic and Health Surveys alongside existing questions on immunization.

### 2. Coordinating Partners

*Each partner wants their own activity to be highest priority for the HBR.* —Key Informant 3

In some respondents' countries, fragmentation and a lack of coordination of the HBR program resulted in the concurrent rollout of multiple records with overlapping content. This was found to inconvenience and confuse families and health care workers alike.

Multiple sources noted that all partners involved in implementing the HBR program needed to frankly discuss their individual priorities at the start of the whole process to ensure they were on the same page and committed to creating harmonious HBR content rather than competing with each other. In the experience of the key respondents, partners could include committees for nutrition, MNCH, immunization, malaria, and breastfeeding, among others, within ministries of health and donor organizations.

Many documents and respondents specified that the overall responsibility for coordinating partners should lie with the ministries of health rather than nongovernmental organizations or donors. They explained that ministries of health usually know their population and health needs better than external groups and having this responsibility helped empower them. It also encouraged the government to eventually assimilate the program into their own budget. This institutionalized the HBR program and gave it greater long-term stability than relying upon donors each year. However, respondents noted that government leadership was difficult to ensure, especially in poorer ministries in which donors exerted more control or within governments in which corruption or lack of knowledge about HBR programs resulted in inaction.

### 3. Selecting HBR Contents

*If it is not being used, be ruthless and take it out. Otherwise, you undervalue the importance of the records.* —Key Informant 10

Sections of the HBR that did not get used at the ground level were noted to not only have been a waste of money but also have been a discouragement to parents and health care workers from using the HBRs altogether. To prevent this, documents and key informants alike noted that contents should have been created and selected appropriately for the local demographics and burdens of disease and for the local health system's available services and staff (for example, the record should not have included sections for recording results of tests that were not available in that country or region).

Contents should have been created and selected appropriately for the local demographics and burdens of disease and for the local health system's available services and staff.

International and ministry-level respondents with policy experience in multiple countries indicated that the HBRs had the dual purposes of recording health parameters and communicating health education messages. However, many respondents with experience in service provision and planning at local levels viewed them only as recordkeeping tools, admitting that any health education content was often overlooked. They suggested emphasizing this dual purpose to health care workers when introducing them to the HBR and training them to use the HBR as a starting point for health education conversations.

Most respondents and a document noted the conflict between choosing single-focus or multiple-focus HBRs. Single-focus HBRs contain content relevant to 1 health topic or 1 population group, for example, vaccination cards for children or antenatal care notes held by pregnant woman.

Multiple-focus HBRs contain content about more than 1 health topic and also have chronologically ordered content that can cover a prolonged period of time. The United Kingdom's National Health Service paper-based child health record—commonly known as the “Red Book”—is a multiple-focus HBR given to a child's caregivers at birth and used for various purposes throughout childhood. It contains vaccination records, growth charts, records of illness, and health education messages. Japan's Maternal and Child Health Handbook, which has also been implemented in many LMICs, contains similar content but also contains antenatal records. The handbook is given to the mother during pregnancy, then after birth, the same handbook is used for the child. In favor of multiple-focus HBRs, some respondents stated that it was simply more convenient for women/caregivers to have a single comprehensive document and that fragmentation into many smaller records had a greater chance of loss. Some respondents with experience of the Maternal and Child Health Handbook also argued that child health HBRs with an antenatal component encouraged mothers/caregivers to be more involved in their child's care. Arguments against multiple-focus HBRs centered upon delays associated with the involvement of multiple partners in creating the different content areas and the increased cost associated with them, which may make them unfeasible in certain settings.

### 4. Designing HBRs

*The problem is that most records are designed top-down.* —Key Informant 9

Interviewees unanimously agreed that the design process should involve community and end user input. They noted that incorporating health care workers' feedback during the design process ensured that the HBR layout aligned with their workflow, making it more likely to be used. In addition, incorporating feedback from the communities that will use the HBR was noted to be vital in ensuring that records were mindful of local cultural sensibilities. Pretesting was consistently mentioned as a vital step in the process to obtain this feedback from end users.

Respondents explained that it was important for regional languages to be represented on HBRs, not just the official or national language/s. Many sources noted the importance of adapting HBRs for low-literacy populations by using pictorials and rigorously testing these to ensure they conveyed the desired message. The role of health care workers in explaining the HBR to a woman or caregiver was also noted to be more significant in low-literacy contexts.

Using pictorials for low-literacy populations and creating HBRs in regional languages or dialects were noted as vital for reaching and engaging end users.

Both respondents and documents indicated that physical durability of the HBR hugely affected its use. A respondent with experience in Nepal noted that their child health cards easily disintegrated in the rain and were so flimsy that they were used as children's drawing paper instead. They piloted a simple plastic cover for the record that increased durability and retention significantly.

Keeping the HBR up-to-date with new best practices and health service changes was noted as vital. Designs and redesigns were found to be most effective when set on a defined timetable, such as biannually. Respondents explained that mandatory HBR redesigns tended to be led by immunization groups when new vaccines entered the national vaccination schedule. One respondent with international experience noted that involving multiple nonimmunization health sectors (whose content is less timebound) could lead to delays, emphasizing the need for partner coordination not only for initial planning stages but for redesigns as well.

### 5. Covering Costs

*A lot of money goes toward design, but when the government asks for funding for training and supervision, suddenly, there is no money left.* —Key Informant 11

International-level respondents and documents described how costs varied with the size of the record, number of topics covered, color versus black-and-white printing, and the system of distribution. However, accurate estimates were not available.

It was reported that costs of HBR programs in LMICs tended to be covered primarily by donors, particularly during early stages of implementation for design, initial printing cycles, and capacity building. Respondents and documents explained that this was often problematic and led to a lack of dependable funding. For example, costs could be covered ad hoc when donors had money to spare at the end of a financial year or only the initial design and the first round of printing would be covered, which caused stock-outs and left the local system struggling to continue the program.

Suggestions for improvement included obtaining long-term donor funding agreements (including contributions from every partner that had inserted their content into the record) each time a batch of HBRs was planned and ensuring the agreement covered all downstream activities, not just design and initial printing. Many sources preferred early planning for a gradual transition to government funding, and they noted that this planning was made easier when the government/Ministry of Health was empowered from the start to lead the process. A few sources mentioned charging women and caregivers for the HBR or covering costs through advertisement space on the record, however, these did not appear to be common practices.

### 6. Printing and Distribution

*Medicines and vaccines have very strong logistics management systems, but HBRs are not currently subject to the same rigor or quality control.* —Key Informant 10

Multiple sources found printing and distribution to be where the most roadblocks occurred. Respondents spoke of how funding often was given for the design and printing of HBRs but not for distribution. They noted that this was a barrier to sustainable HBR programs and a major cause of stock-outs.

A solution proposed by some sources was to integrate HBRs into existing health system supply and distribution chains just like vaccines, medicines, and other essential health commodities. However, they cautioned that in many settings, these existing chains also required strengthening. Respondents explained that efficient printing and distribution required planning ahead and accurately estimating the demand for HBRs. This planning was usually performed yearly by district health management teams using population and birth data, which were incomplete in some settings. One respondent noted that rural and remote regions and those with large transient populations (e.g., refugees) posed particular challenges both in terms of estimating numbers and in terms of HBRs and other services practically reaching them.

Efficient printing and distribution required planning ahead and accurately estimating the demand for HBRs.

### 7. Promoting Use of HBRs Among Health Care Providers

*There's an assumption that training health care providers at the national level on how to use the records will be enough to get them to use them. That doesn't work in reality. Providers need on-site mentoring and coaching to use the records to habituate them.* —Key Informant 4

A commonly cited barrier to effective HBR programs was that health care workers did not always know or care enough about using the records. Two respondents explained that an underlying reason for lack of motivation to engage with the HBRs was a lack of understanding of their value. HBRs were created primarily to record health data, but some HBRs also function to promote health education. Many respondents and documents alike praised these dual-purpose HBRs. However, we learned that in reality, they were rarely used as a deliberate starting point for conversations between health care workers and women or caregivers. Sources explained that health care workers were often unaware of the educational purpose and its importance. They also suggested that that these issues could be due to time-restricted consultations and a sense that completing HBRs was double the work because most health care workers had to complete their own facility-based records as well.

A commonly cited barrier to effective HBR programs was that health care workers did not always know or care enough about using the records.

For a more successful HBR program, sources explained that initial training on using the HBR must be given at the local level to all health care workers in both the public and private sectors who deal with HBRs. Centralized training for only senior health care workers was deemed insufficient. They also explained that initial training for health care workers was paramount, but ongoing refresher training, which was often neglected, was equally important. Refresher training was especially useful for community health workers who tended to have less formal education but who were vital in engaging rural or disenfranchised women/caregivers with the HBR. One suggestion for legitimizing HBRs was to mandate HBR training as part of preservice professional training and as a prerequisite for professional body accreditation. Another suggestion was to reward health care workers who complete the most HBRs in practice.

### 8. Promoting Use of HBRs Among Pregnant Women and Parents/Caregivers of Children

*Women like records. They make them feel important, like they are taking care of themselves, and like the provider will take more notice of them if they bring them.* —Key Informant 4

Respondents stated several factors that they believed affected HBR use and retention by pregnant women and children's caregivers. Education was noted to improve understanding of the purpose of the records, and urban living was noted to be associated with lower rates of HBR loss due to shorter travel distances over less difficult terrain. Having a clear and consistent policy for replacement of lost or damaged records was also noted to be important.

Many documents and nearly all respondents spoke about the importance of patients/caregivers being introduced carefully to the HBR when they received it for the first time, with an explanation of its value and the necessity of bringing it to every health visit. Respondents reported that in some countries, HBR-documented proof of vaccination completion was required for school entry, and in others, HBRs were used to officially register a child's birth. These measures were thought to increase the legitimacy and value of the HBR. Involving health professionals, such as community health workers and midwives, and nonhealth-sector stakeholders, such as religious and community leaders, in HBR pretesting and promotion was also viewed as important for gaining the acceptance of end users and making HBRs more relevant to them.

When patients/caregivers receive the HBR for the first time, they should be given an explanation of its value and of the necessity of bringing it to every health visit.

## DISCUSSION

This framework analysis formed the final study in a WHO-commissioned series to inform the 2018 WHO recommendations on HBRs in MNCH.^3^ Our findings indicated 8 key components that played a crucial role in determining successful implementation of HBRs. These were relevant to those involved with HBRs at all levels: ministry and policy, program, and end user.

We hope that the findings will help health care workers, pregnant women, and parents/caregivers of children use HBRs more effectively, provide key program stakeholders with a rough blueprint of the implementation process to be adapted for individual regional contexts when beginning and/or strengthening implementation, and help health care providers and planning groups identify and develop solutions for roadblocks in their own HBR programs.

Our findings build on the existing body of literature on HBR implementation. Studies found that mandating HBR ownership for all children in Indonesia^27^ or requiring proof of immunization for school entry in Australia^28^ both led to increased HBR ownership and retention. Conversely, when the government's agenda priorities for HBRs were not articulated into clear policies or incentives, various providers interpreted these priorities differently, resulting in a lack of consistent progress.^29^

Our respondents suggested including questions about HBRs in Demographic and Health Surveys and censuses to build credible advocacy for high-level support. However, analysis showed that a question on HBR ownership has been already included in the Demographic and Health Surveys for the majority of countries.^13^ Perhaps to garner more high-level support, the Demographic and Health Surveys should include more specific questions to qualify patterns of HBR use or should adapt the way the data are harnessed to turn it into constructive action and advocacy.

The need for coordination of partners came up often in our data. Unfortunately, no research currently exists specifically on how to coordinate donors, nongovernmental organizations, and ministry departments within a country who are all working on the same HBR program, but it was recognized as necessary to prevent fragmentation of HBRs and services.^30^ However, there is evidence that at the regional level, there has been coordination between LMICs to visualize and prototype improvements in their HBRs while focusing on their country-specific contexts.^31^ This may remain difficult to operationalize due to crowded agendas, limited resources, and the priorities of donor organizations.^31^

Many HBR content areas were not used by end users and not referred to by health care providers during consultations.^14^ In multiple-focus child health or maternal and child health books, the patient demographics and vaccination sections were the most consistently completed sections, and other sections including the growth monitoring charts were often neglected.^32^ As our respondents alluded, having unused content sections could undermine the value of the rest of the HBR as perceived by both health care workers and women or caregivers. The extra resources used in creating these unused sections may have been unjustifiable and unnecessary costs.^14^ Reasons for unused content must be explored, and when similar HBR formats are being implemented in multiple countries, the content should be adapted to be country- and region-specific.^33^

There was insufficient evidence to recommend one form of HBR over others, and no studies that directly compared the implementation of a multiple-focus HBR to a single-focus HBR. Multiple-focus HBRs, for example, maternal and child health handbooks, were widely recognized to be more expensive to print.^5^^,^^33^ A theoretical argument suggested that this was more cost-effective overall than printing multiple single-focus records;^33^^,^^34^ however, formal economic evaluation is required. The choice of single-focus or multiple-focus HBR is likely to be context-specific, so more local research in LMICs is required to delineate these context-specific determinants.^3^^,^^25^

A number of existing studies have affirmed the importance of the design of the HBR.^16^^–^^19^ In addition, in 2015, the WHO published a guide for designing, using, and promoting HBRs in immunization programs.^2^ All these sources advocate for plastic covers for physical durability, simple uncluttered layout, large print size, nontechnical language, attractive colors and shading, and clear photos or illustrations. Incorporating these changes when redesigning immunization cards contributed to improving childhood immunization adherence in studies in both urban and rural Pakistan.^18^^,^^19^

Our sources echoed existing studies advocating that HBRs should be created in regional languages. For example, the Road to Health card in South Africa was printed in English, Afrikaans, and Xhosa,^21^ and the Patient Passport in the USA had better impact when there was a concurrent Spanish version.^35^ An informal review conducted by Brown showed that the health messages in HBRs were often poorly aligned to national literacy levels.^25^ Studies agreed that clear pictorials were needed for women and caregivers with low-literacy levels, but even these could sometimes be difficult to decipher, and they often required additional support from health care workers or relatives to access the HBR content.^20^^,^^36^^,^^37^ This could pose concerns regarding equity in accessing the HBR content and needs to be explored further.

There was a substantial theoretical case for the long-term health system cost savings associated with an effective HBR program.^38^ Regarding current funding of HBR programs, a survey of immunization records in 135 countries showed that the majority of HBR financing was shared between the health ministry and development partners or other partnership combinations.^5^ There was greater involvement of development partners in low-income countries and those in the GAVI alliance.^5^ Relying solely on single-source funding arrangements seemed to be more associated with HBR stock-outs, as with very complex multi-source funding arrangements.^39^ More research on the funding arrangements of all types of HBR programs—building on existing research on vaccination cards—is vital to be able to better forecast demand and help build sustainable funding streams for each component of the implementation process.

As our data showed, the systems for estimating demand and printing HBRs accordingly were often poorly planned and maintained. In data from WHO and UNICEF's Joint Reporting Form on Immunization, 48 of 194 countries reported at least 1 national level HBR stock-out during a 3-year period. Several of these countries had multiple stock-outs. In addition, the supply chains were often poorly monitored: 75 countries did not have any information about HBR stock-outs in at least 1 of the 3 years.^39^

Even when an adequate number of HBRs are printed, they must reach the appropriate end users, including those who are remote or disenfranchised. In a study of slum communities in Kampala, Uganda, delivering in a health facility was found to be the strongest determinant of receiving an HBR. However, the majority of births in Uganda occurred outside formal health facilities.^40^ This raises the challenge of how to extend HBR distribution chains and to incorporate HBRs into existing health system strengthening programs.

There was evidence to suggest that health care workers in low-income settings valued HBRs, and especially integrated multi-focus HBRs, for the information provided, convenience, and long-term value.^7^ This was especially true in settings where facility-based record systems were incomplete or incorrect. In addition, studies indicated that HBRs that incorporated not only the data recording function but also the additional health education function provided a common talking point for patients and health care workers, enhancing patient-provider communication.^7^ In contrast, our sources felt that in some settings, enhanced communication was an ideal rather than a reality due to various barriers preventing health care workers from realizing the value of HBRs. For example, health care workers did not use or promote HBRs well when the contents did not match their workflow. Therefore, they must be involved in the content selection and design process.^21^

There was evidence that overall, women and caregivers valued HBRs for recording information, increasing their knowledge about their or their child's health care, and increasing their sense of empowerment during interactions with health professionals to enable shared decision making.^7^ However, this value was not necessarily automatic or inherent. Studies across multiple countries agreed with our respondents that the health care worker's initial explanation and request to see the HBR at every health visit determined the owner's perception of their HBR's value and their subsequent engagement with it. Retention of the record plummeted when the record was not explained clearly.^22^^–^^24^^,^^32^

Strengths of this study include a broad and comprehensive approach to exploring HBR implementation; this meant that we were able to develop a more complete picture of the implementation process, not simply 1 or 2 factors. Our key respondents had a breadth of experience: across continents, across different health and development sectors, and involving both policy- and ground-level implementation. They had experience predominantly in LMICs. In contrast, the majority of existing HBR literature was from high-income countries. Finally, in this framework analysis, we reached saturation on the major themes (i.e., minimal additional information emerged from subsequent interviews), and there was strong consistency between interview respondents and gray literature documents.

This study used a broad and comprehensive approach to develop a more complete picture of the HBR implementation process.

Further research is required on the perspectives and motivations of health care workers who deal with HBRs and on how HBRs can be effectively integrated with existing facility-based health information systems. Research is also required on the differences between implementing single-focus and multiple-focus HBRs, including whether their unique characteristics make them suited to different contexts or populations. Accurate costings of the full implementation process are needed, including for downstream activities such as printing and distribution, to enable health ministries and donors to make informed and sustainable planning decisions.

The implementation components that we have identified come from experiences of successes and failures in HBR programs. Some of the strategies suggested are mirrored in the existing literature, but some are new and require further research on how they can best be operationalized. There are multiple interlinkages between our 8 implementation components. For example, high-level support and partner coordination is required to secure long-term funding, which determines the potential for designs and redesigns, printing, distribution, and initial as well as refresher training for health care providers. Meanwhile, the content areas chosen and the design of the HBR affect how it is valued and used by health care providers and pregnant women/caregivers.

We can assume that if all the 8 components are optimized, an HBR program will be more successful. However, more operational research is required to confirm this, to understand how much each individual factor contributes, and to explore how they interact with each other in different country contexts.

### Limitations

This study had several limitations. We had a small number of respondents (n=12). Some of our 12 respondents had hands-on, frontline experience with HBRs, but we were directed toward these country-level respondents by international-level respondents at donor organizations. Secondly, although key respondents talked about their experience with failures in HBR programs, the gray literature rarely covered this because, unfortunately, donor-published reports were seldom willing to publicize failures.

In addition, a few subthemes did not reach saturation since they were only addressed briefly or by only 1 or 2 sources. These subthemes included electronic HBRs and the use of HBRs in vulnerable populations such as refugees. One respondent with experience in refugee settings described meeting a mother who had arrived after an arduous journey to the country of asylum with very few essential and valuable items, one of which was her infant's HBR. The WHO recommendations on HBRs note that transient and vulnerable populations in particular stand to benefit from records that they can carry with them.^3^ Meanwhile, electronic records have been well studied in the literature, though almost exclusively in high-income countries. Implementation barriers included lack of universal high-speed internet access, concerns about data confidentiality, complexity of software, and difficulties integrating electronic HBRs with facility records.^41^^–^^45^ The implementation of electronic records in LMICs that have just started to pilot electronic HBRs and are rapidly growing in technological capabilities deserves attention in future research.

## CONCLUSION

Home-based records are a remarkably diverse group of tools that can be complex to implement and coordinate. They already exist worldwide, albeit with inconsistent coverage, but have been neglected when it comes to research, quality improvement, and funding. Our findings provide a more comprehensive overview of the implementation process for HBR programs. The hope for the related WHO guidelines^2^ is that policy makers, donors, and the end users of HBRs (frontline health care providers, pregnant women, and the families of children) will all be able to better harness the benefits of HBRs for improved MNCH outcomes, increased participation and shared decision making, and better coordination and continuity of care.

## Supplementary Material

19-00340-Mahadevan-Supplement_Material.pdf

## Notes

**Peer Reviewed**

**First published online:** March 11, 2020

**Funding:** Funding was provided by the World Health Organization Department of Maternal, Newborn, Child and Adolescent Health (now the Department of Maternal, Newborn, Child and Adolescent Health and Aging) through a grant from the Japan International Cooperation Agency.

## References

[1] Home-Based Record Repository. 2017. http://www.immunizationcards.org/. Accessed August 10, 2019.

[2] World Health Organization (WHO). Practical Guide For the Design, Use and Promotion of Home-Based Records In Immunization Programmes. Geneva: World Health Organization; 2015. https://apps.who.int/iris/bitstream/handle/10665/175905/WHO_IVB_15.05_eng.pdf?sequence=2. Accessed March 6, 2019.

[3] World Health Organization (WHO). WHO Recommendations on Home-Based Records for Maternal, Newborn and Child Health. Geneva: World Health Organization; 2018. https://apps.who.int/iris/bitstream/handle/10665/274277/9789241550352-eng.pdf?ua=1. Accessed December 1, 2018. 30325618

[4] Brown DW Gacic-Dobo M Young SL. Home-based child vaccination records – A reflection on form. Vaccine. 2014;32(16):1775–1777. 10.1016/j.vaccine.2014.01.098. 24530931

[5] Young SL Gacic-Dobo M Brown DW. Results from a survey of national immunization programmes on home-based vaccination record practices in 2013. Int Health. 2015;7(4):247–255. 10.1093/inthealth/ihv014. 25733540 PMC4492340

[6] Magwood O Kpadé V Thavorn K Oliver S Mayhew AD Pottie K. Effectiveness of home-based records on maternal, newborn and child health outcomes: a systematic review and meta-analysis. PLoS One. 2019;14(1):e0209278. 10.1371/journal.pone.0209278. 30601847 PMC6314587

[7] Magwood O Kpadé V Afza R. Understanding women's, caregivers', and providers' experiences with home-based records: a systematic review of qualitative studies. PLoS One. 2018;13(10). 10.1371/journal.pone.0204966. 30286161 PMC6171900

[8] Mori R Yonemoto N Noma H. The Maternal and Child Health (MCH) handbook in Mongolia: a cluster-randomized, controlled trial. PLoS One. 2015;10(4):e0119772. 10.1371/journal.pone.0119772. 25853511 PMC4390384

[9] Osaki K Hattori T Toda A. Maternal and Child Health Handbook use for maternal and child care: a cluster randomized controlled study in rural Java, Indonesia. J Public Health (Oxf). 2019;41(1):170–182. 10.1093/pubmed/fdx175. 29325171 PMC6459363

[10] Elbourne D Richardson M Chalmers I Waterhouse I Holt E. The Newbury Maternity Care Study: a randomized controlled trial to assess a policy of women holding their own obstetric records. Br J Obstet Gynaecol. 1987;94(7):612–619. 10.1111/j.1471-0528.1987.tb03165.x. 3304403

[11] World Health Organization. Framework on integrated people-centred health services. Report by the Secretariat. Geneva, Switzerland: World Health Organization; 2016. http://apps.who.int/gb/ebwha/pdf_files/WHA69/A69_39-en.pdf?ua=1&ua=1. Accessed April 24, 2019.

[12] Bauer MS Damschroder L Hagedorn H Smith J Kilbourne AM. An introduction to implementation science for the non-specialist. BMC Psychol. 2015;3(1):32. 10.1186/s40359-015-0089-9. 26376626 PMC4573926

[13] Brown DW Gacic-Dobo M. Home-based record prevalence among children aged 12–23 months from 180 Demographic and Health Surveys. Vaccine. 2015;33(22):2584–2593. 10.1016/j.vaccine.2015.03.101. 25887089

[14] Brown DW Bosch-Capblanch X Shimp L. Where do we go from here? Defining an agenda for home-based records research and action considering the 2018 WHO guidelines. Glob Health Sci Pract. 2019;7(1):6–11. 10.9745/ghsp-d-18-00431. 30877139 PMC6538131

[15] World Health Organization (WHO). Improving the Quality of Health Services - Tools and Resources. Turning Recommendations into Practice. Geneva, Switzerland: WHO; 2018. https://apps.who.int/iris/bitstream/handle/10665/310944/9789241515085-eng.pdf. Accessed December 19, 2019.

[16] Shah PM Selwyn BJ Shah K Kumar V. Evaluation of the home-based maternal record: a WHO collaborative study. Bull World Health Organ. 1993;71(5):535–548. 8261557 PMC2393478

[17] Lakhani AD Avery A Gordon A Tait N. Evaluation of a home based health record booklet. Arch Dis Child. 1984;59(11):1076–1081. 10.1136/adc.59.11.1076. 6508341 PMC1628817

[18] Usman HR Akhtar S Habib F Jehan I. Redesigned immunization card and center-based education to reduce childhood immunization dropouts in urban Pakistan: a randomized controlled trial. Randomized Controlled Trial. 2009;27(3):467–472. 10.1016/j.vaccine.2008.10.048. 18996423

[19] Usman HR Rahbar MH Kristensen S. Randomized controlled trial to improve childhood immunization adherence in rural Pakistan: redesigned immunization card and maternal education. Trop Med Int Health. 2011;16(3):334–342. 10.1111/j.1365-3156.2010.02698.x. 21159080 PMC3763701

[20] World Health Organization (WHO). Home Based Maternal Records: Guidelines for Development, Adaptation and Evaluation. Geneva, Switzerland: WHO; 1994.

[21] Harrison D Harker H Hesse Hd Mann MD. An assessment by nurses and mothers of a ‘road-to-health’ book in the Western Cape. Curationis. 2005;28(4):57–64. 10.4102/curationis.v28i4.1021. 16450560

[22] Hawley G Hepworth J Wilkinson SA Jackson C. From maternity paper hand-held records to electronic health records: what do women tell us about their use? Aust J Prim Health. 2015;22(4):339–348. 10.1071/py14170. 26351241

[23] Hamilton L Wyver S. Parental use and views of the child personal health record. Educ Dev Psychol. 2012;29(1):66–77. 10.1017/edp.2012.2

[24] Pahari DP Bastola SP Paudel R. Factors affecting retention of child health card in a rural area. J Nepal Health Res Counc. 2011;9(2):154–158. 22929845

[25] Brown DW. Additional considerations for maternal and child health handbooks in light of WHO's recommendations on home-based records for maternal, newborn and child health: a commentary in response to Nakamura. J Glob Health Sci. 2019;1(2). 10.35500/jghs.2019.1.e32

[26] Gale NK Heath G Cameron E Rashid S Redwood S. Using the framework method for the analysis of qualitative data in multi-disciplinary health research. BMC Med Res Methodol. 2013;13(1):117. 10.1186/1471-2288-13-117. 24047204 PMC3848812

[27] Osaki K Hattori T Kosen S Singgih B. Investment in home-based maternal, newborn and child health records improves immunization coverage in Indonesia. Trans R Soc Trop Med Hyg. 2009;103(8):846–848. 10.1016/j.trstmh.2009.03.011. 19375141

[28] Jeffs D Nossar V Bailey F Smith W Chey T. Retention and use of personal health records: A population-based study. J Paediatr Child Health. 1994;30(3):248–252. 10.1111/j.1440-1754.1994.tb00627.x. 8074911

[29] Essén A Gerrits R Kuhlmann E. Patient accessible electronic health records: Connecting policy and provider action in the Netherlands. Health Policy Technol. 2017;6(2):134–141. 10.1016/j.hlpt.2017.03.001

[30] Aiga H Nguyen VD Nguyen CD Nguyen TT Nguyen LT. Fragmented implementation of maternal and child health home-based records in Vietnam: need for integration. Glob Health Action. 2016;9(1):29924. 10.3402/gha.v9.29924. 26928218 PMC4770865

[31] Hasman A Rapp A Brown DW. Revitalizing the home-based record: reflections from an innovative south-south exchange for optimizing the quality, availability and use of home-based records in immunization systems. Vaccine. 2016;34(47):5697–5699. 10.1016/j.vaccine.2016.09.064. 27743647

[32] Brown DW Tabu C Sergon K. Home-based record (HBR) ownership and use of HBR recording fields in selected Kenyan communities: results from the Kenya Missed Opportunities for Vaccination Assessment. PLoS One. 2018;13(8):e0201538. 10.1371/journal.pone.0201538. 30071060 PMC6072064

[33] Nakamura Y. The role of maternal and child health (MCH) handbook in the era of sustainable development goals (SDGs). J Glob Health Sci. 2019;1(1):e24. 10.35500/jghs.2019.1.e24

[34] Aiga H Pham Huy TK Nguyen VD. Cost savings through implementation of an integrated home-based record: a case study in Vietnam. Public Health. 2018;156:124–131. 10.1016/j.puhe.2017.12.018. 29427768

[35] Lee LK Mulvaney-Day N Berger AM Bhaumik U Nguyen HT Ward VL. The Patient Passport Program: an intervention to improve patient-provider communication for hospitalized minority children and their families. Acad Pediatr. 2016;16(5):460–467. 10.1016/j.acap.2015.12.008. 26724179

[36] Yanagisawa S Soyano A Igarashi H Ura M Nakamura Y. Effect of a maternal and child health handbook on maternal knowledge and behaviour: a community-based controlled trial in rural Cambodia. Health Policy Plan. 2015;30(9):1184–1192. 10.1093/heapol/czu133. 25595142 PMC4597043

[37] Hagiwara A Ueyama M Ramlawi A Sawada Y. Is the Maternal and Child Health (MCH) handbook effective in improving health-related behavior? Evidence from Palestine. J Public Health Policy. 2013;34(1):31–45. 10.1057/jphp.2012.56. 23151920

[38] Brown DW. A common sense business case for investing in a revitalization of home-based records within immunization delivery systems. White Paper Version. 2017. Accessed December 10, 2019. 10.13140/RG.2.2.32537.42082

[39] Brown DW Gacic-Dobo M. Occurrence of home-based record stock-outs—a quiet problem for national immunization programmes continues. Vaccine. 2018;36(6):773–778. 10.1016/j.vaccine.2017.12.070. 29307476 PMC5789753

[40] Mukanga D Kiguli S. Factors affecting the retention and use of child health cards in a slum community in Kampala, Uganda, 2005. Matern Child Health J. 2006;10(6):545–552. 10.1007/s10995-006-0132-9. 16850275

[41] Byczkowski TL Munafo JK Britto MT. Family perceptions of the usability and value of chronic disease web-based patient portals. Health Informatics J. 2014. 20(2):151–162. 10.1177/1460458213489054. 24056751

[42] Fisher B. Patient record access: making it work for you and the NHS. Lond J Prim Care (Abingdon). 2011;4(1):43–48. 10.1080/17571472.2011.11493327. 25949647 PMC3960663

[43] Fuji KT Abbott AA Galt KA. Personal Health Record Design: Qualitative Exploration of Issues Inhibiting Optimal Use. Diabetes Care. 2014;37(1):e13–e14. 10.2337/dc13-1630. 24356602 PMC3968449

[44] O'Connor S Devlin AM McGee-Lennon M Bouamrane MM O'Donnell CA Mair FS. Factors affecting participation in the eRedBook: a personal child health record. Stud Health Technol Inform. 2016;225:971–972. 10.3233/978-1-61499-658-3-971. 27332437

[45] Weitzman ER Kaci L Mandl KD. Acceptability of a personally controlled health record in a community-based setting: implications for policy and design. J Med Internet Res. 2009;11(2):e14. 10.2196/jmir.1187. 19403467 PMC2762802

