# Supplementary material for: How Should Home-Based Maternal and Child Health Records Be Implemented? A Global Framework Analysis
Source: Glob Health Sci Pract. 2020 Mar 30;8(1):100–13. doi: 10.9745/GHSP-D-19-00340 (PMC7108936; doi:10.9745/GHSP-D-19-00340)
Supplement: 19-00340-Mahadevan-Supplement_Material.pdf [file 19-00340-Mahadevan-Supplement_Material.pdf]

Supplement to: Mahadevan S, Broaddus-Shea E. How Should Home-Based Maternal and Child Health Records Be Implemented? A Global Framework Analysis. *Glob Health Sci Pract*. 2020;8(1). https://doi.org/10.9745/GHSP-D-19-00340

Supplement: Initial and Final Framework

Initial Framework Components Based on Subject Expert Inputs

Category	Topic/Theme	Description
Descriptive metadata	Source	Title of article/document or interviewee's organization and position/qualifications
	Nature of evidence	Type of data presented (e.g., key-informant interview, qualitative study, program report, country workshop report, workshop summary report)
	Type of record	Information gathered by HBR described by data source (e.g., vaccinations, child health, maternal health).
	Location	Specific country or region addressed by data source
	Setting	Any descriptive details about the implementation setting noted by data source
Contextual factors	Urbanization	Ways in which dense urban settings, peri-urban settings, or remote rural settings impact HBR implementation
	Literacy	Ways in which levels of literacy within a population impact HBR implementation
	Societal norms	Ways in which societal norms (particularly constrictive norms) impact HBR implementation
	Health systems	Ways in which aspects of a setting's health system (particularly methods of service delivery, reliance on outreach services, and level of centralization) impact HBR implementation
Implementation parameters:	Approval of records/redesigns	Recommendations for or challenges noted regarding who leads and is involved in these processes and how approvals are assured

Supplement to: Mahadevan S, Broaddus-Shea E. How Should Home-Based Maternal and Child Health Records Be Implemented? A Global Framework Analysis. *Glob Health Sci Pract*. 2020;8(1). https://doi.org/10.9745/GHSP-D-19-00340

Category	Topic/Theme	Description
administrative	Coordination of partners	Recommendations for or challenges noted regarding coordination of partners within the government as well as external partners
	Decentralization	Recommendations for or challenges noted regarding decentralization to districts and provinces
	Training of frontline health care providers	Recommendations for or challenges noted regarding training of providers
	Funding	Recommendations for or challenges noted regarding funding of printing, distribution and resupply, and training, as well as any discussion of funding through government budget lines vs. external funding
	Cost	Any discussion of costs associated with aspects of implementation and who covers them
Implementation parameters: logistics	Printing	Recommendations for or challenges noted regarding availability of quality, reliable print services, and/or any discussion of the market shaping printing services at regional or subregional levels
	Distribution and resupply to frontline	Recommendations for or challenges noted regarding distribution and resupply
	Introduction and interpersonal communication with mothers/caregivers	Recommendations for or challenges noted regarding communication with mothers/caregivers, particularly relating to card retention/value

Category	Topic/Theme	Description
	Replacement policies/practices	Recommendations for or challenges noted regarding replacement policies and practices
	Stock management	Recommendations for or challenges noted regarding assuring stock management and availability over several years
Implementation parameters: materials	Design	Recommendations for or challenges noted regarding design, including features of added value such as certificates of graduation/completion of infant vaccination series
	Durability	Recommendations for or challenges noted regarding durability of record materials, including a discussion of the market shaping durable paper options at regional or subregional levels

Supplement to: Mahadevan S, Broaddus-Shea E. How Should Home-Based Maternal and Child Health Records Be Implemented? A Global Framework Analysis. *Glob Health Sci Pract*. 2020;8(1). https://doi.org/10.9745/GHSP-D-19-00340

Final Revised Framework

Implementation Components	Key Themes Identified in Data
Establishing high-level support	• Importance of prioritizing HBRs<br>• Need for advocacy and education<br>• Importance of data about HBR usage and outcomes
Coordinating partners	• Importance of government-led coordination<br>• Ensuring alignment with health system structures, guidelines, and capacity<br>• Need for balanced representation across sectors<br>• Establishing consensus among partners on record contents and use
Selecting HBR contents	• Different understandings of HBR purpose<br>• Benefits and drawbacks of multidomain HBRs<br>• Importance of considering local context, end use, and implementation
Designing HBRs	• Influence of design on use<br>• Need for end user (both health care provider and caregiver) input during design process<br>• Meeting the needs of those with low literacy<br>• Consideration of regional and local languages<br>• Efficiently carrying out redesigns when needed
Covering costs	• Ensuring adequate and dependable funding<br>• Transitioning from donor to government funding
Printing and distribution	• Importance of integrating HBRs into health system supply chains<br>• Need for planning, accurate demand prediction, and oversight
Promoting use of HBRs among health care providers	• Need for motivation and engagement<br>• Challenges with ensuring effective record completion and communication with patients/caregivers<br>• Need for ongoing monitoring and coaching<br>• Importance of reaching out to private sector and secondary care facilities
Promoting use of records among patients/caregivers	• Determinants of valuing and retaining HBRs<br>• Need for thorough introduction and explanation of HBRs

Implementation Components	Key Themes Identified in Data
	• Importance of community health workers and community engagement
